# Characterization of Binding of Magnetic Nanoparticles to Rolling Circle Amplification Products by Turn-On Magnetic Assay

**DOI:** 10.3390/bios9030109

**Published:** 2019-09-17

**Authors:** Sobhan Sepehri, Björn Agnarsson, Teresa Zardán Gómez de la Torre, Justin F. Schneiderman, Jakob Blomgren, Aldo Jesorka, Christer Johansson, Mats Nilsson, Jan Albert, Maria Strømme, Dag Winkler, Alexei Kalaboukhov

**Affiliations:** 1 Department of Microtechnology and Nanoscience—MC2, Chalmers University of Technology, SE-412 96 Göteborg, Sweden; 2 Department of Physics, Chalmers University of Technology, SE-412 96 Göteborg, Sweden; 3 Department of Engineering Sciences, Uppsala University, The Ångström Laboratory, Box 534, SE-751 21 Uppsala, Sweden; 4 MedTech West and the Institute of Neuroscience and Physiology, University of Gothenburg, SE-405 30 Göteborg, Sweden; 5 RISE—Research Institutes of Sweden, SE-411 33 Göteborg, Sweden; 6 Department of Chemistry and Chemical Engineering, Chalmers University of Technology, SE-412 96 Göteborg, Sweden; 7 Science for Life Laboratory, Department of Biochemistry and Biophysics, Stockholm University, Box 1031, SE-171 21 Solna, Sweden; 8 Department of Clinical Microbiology, Karolinska University Hospital, SE-171 76 Stockholm, Sweden; 9 Department of Microbiology, Tumor and Cell Biology, Karolinska Institute, SE-171 77 Stockholm, Sweden

**Keywords:** magnetic nanoparticle, bioassay, differential homogenous magnetic assay, immobilization, binding kinetics, rolling circle amplification product

## Abstract

The specific binding of oligonucleotide-tagged 100 nm magnetic nanoparticles (MNPs) to rolling circle products (RCPs) is investigated using our newly developed differential homogenous magnetic assay (DHMA). The DHMA measures ac magnetic susceptibility from a test and a control samples simultaneously and eliminates magnetic background signal. Therefore, the DHMA can reveal details of binding kinetics of magnetic nanoparticles at very low concentrations of RCPs. From the analysis of the imaginary part of the DHMA signal, we find that smaller MNPs in the particle ensemble bind first to the RCPs. When the RCP concentration increases, we observe the formation of agglomerates, which leads to lower number of MNPs per RCP at higher concentrations of RCPs. The results thus indicate that a full frequency range of ac susceptibility observation is necessary to detect low concentrations of target RCPs and a long amplification time is not required as it does not significantly increase the number of MNPs per RCP. The findings are critical for understanding the underlying microscopic binding process for improving the assay performance. They furthermore suggest DHMA is a powerful technique for dynamically characterizing the binding interactions between MNPs and biomolecules in fluid volumes.

## 1. Introduction

Rolling circle amplification (RCA) is a versatile tool with applications in nanobiotechnology, diagnostics and biodetection [1]. It has high specificity that is ideal for high resolution sequence detection and is an isothermal process that is simple to implement and integrate into microsystems for point-of-care diagnostic [2]. The RCA products contain multiple repetitive complementary copies of a circular DNA template which can be customized to have different functional sequences [1]. For detection of nucleic acids, the assay uses padlock probe ligation [3] for target recognition and RCA [4] to create a single strand concatemer, i.e., the rolling circle product (RCP). The RCPs can be analysed by various methods including gel electrophoresis [5,6], incorporation of fluorophore-conjugated dNTP for fluorescent microscopy and spectroscopy [7] or hybridization of complementary strands with fluorescence markers, gold nanoparticles [8], magnetic nanoparticles (MNPs) [9], quantum dots [10], etc.

MNPs are particularly promising tags due to their high surface-to-volume ratio, biocompatibility, physical and chemical stability, and they can be easily manipulated using an external magnetic field [11,12]. In addition, biological samples are generally not magnetic and thus do not affect the sensitivity/signal transduction in the magnetic domain, unlike how fluorescence can in the optical domain. Numerous methods have been developed for sensing the magnetically labelled target analytes in a volume [13] and on a sensor surface and some of which are used for detection of magnetically labeled RCPs including: magneto-resistive sensors [14], superconducting quantum interference devices (SQUIDs) [9,15,16,17], induction coils [18,19], optomagnetic sensors [20,21], and ferromagnetic resonance sensors [22]. The MNPs are conjugated to the RCPs by base pare hybridization forming MNP-RCP complexes which have a larger hydrodynamic size compared to the unbound MNPs. The increase in the hydrodynamic size changes the characteristic Brownian relaxation dynamics of the MNP-RCP complexes, c.f. Section 4.4. The MNP-RCP ensemble therefore has two relaxation dynamics and consequently two relaxation frequencies: (I) a high relaxation frequency (HRF) which is characteristic of the unbound MNPs and (II) a low relaxation frequency (LRF) which is a result of the MNP-RCP complexes. The change in the hydrodynamic size due to binding to target can be measured using magnetic ac susceptibility [23,24]. This size change appears as a shift in the susceptibility curve. However, due to broad size distribution of the MNPs it is difficult to measure the frequency shift and magnetic assays based on this method have low sensitivity.

In another approach, MNPs are bound to large RCPs (or agglomerate due to association with target analyte) which significantly changes their relaxation frequency. In this case, the binding is detected as a reduction in the HRF peak amplitude at the frequency corresponding to the free (unbound) MNPs, turn-off detection [9,25]. The turn-off detection approach has been used for quantification of magnetically labelled RCPs using various kinds of sensors such as induction coils [26], optomagnetic [27], SQUID [15,17]. The main issue with this approach is the magnetic background from the unbound MNP markers in the solution. At very low concentration of RCPs, very small fraction of the available markers in the sample solution is consumed by forming MNP-RCP complexes. The population of these complexes are basically too small compared to the unbound MNP markers background to produce any significantly detectable signal in the ac susceptibility. This imposes the MNP concentration to be close to the RCP one in order to produce a measurable fraction of bound MNPs. An alternative approach is a turn-on detection strategy [28,29] where an increase of LRF peak amplitude as a result of MNPs binding to the RCPs is measured. Attempts have been made to optimize the magnetic assay by using the small changes observed at low frequencies in the 2nd order harmonic components in the optomagnetic sensor [29]; however, since this approach is more in the line with traditional turn-off assays, it fails to provide real-time data. Since the size of the MNPs is usually comparable to the RCPs, the MNPs cannot bind to the inner side of the RCPs and the number of MNPs that can hybridize to the RCPs is therefore limited. Using the turn-off approach, it has been shown that the number of bound MNPs mainly depends on the amplification time (which determines the size of the RCPs), the size of the MNPs [28,30], and the oligonucleotide surface coverage of the MNPs [30]. While optical methods such as fluorescent microscopy or dynamic light scattering are used to obtain information about the size distribution of the MNP-RCP complexes, the turn-off method is unable to provide similar information and further it fails to determine the LRF from the MNP-RCP complexes for MNPs larger than 40 nm at RCP concentration levels that are of any practical use [28]. This is mainly due to the large magnetic background from the unbound MNPs which obscures the LRF signal.

Recently we developed a differential homogenous magnetic assay (DHMA) which measures a test and a control sample simultaneously to eliminate the troublesome magnetic background [31]. This enables the subtraction of signal from the excess unbound MNPs, which is analogous to a physical washing step in most bulk assays for removing excess markers. In this way, the DHMA is able to resolve directly the very small differences in the nanoparticle’s concentrations and size distributions in the two samples. Using the assay we already demonstrated fM sensitivity with a constant 50% binding fraction for all low concentrations of target molecules [31]. The 50% binding fraction ensures that the MNP markers do not mask the LRF. The DHMA is therefore a turn-on detection strategy which detects small changes in the MNP size distribution by measuring the LRF of the MNP-RCP complex and the HRF of the unbound MNPs.

Here, we use DHMA to investigate the binding characteristics of 100 nm MNPs to RCPs using both turn-off and turn-on detection approaches, see Figure S1. The DHMA clearly reveals the presence of two distinct relaxation processes, LRF and HRF, which can be obtained even at very low concentrations of the RCPs. This has never been seen in the turn-off detection approach. The signal is studied for RCPs of different concentrations and amplification times. The concentration of the RCPs affects the number of MNPs per RCPs due to formation of MNP-RCP agglomerates. Nanoparticle tracking analysis (NTA) is also used to provide information regarding the size distribution of the RCPs. To better understand the binding mechanism of MNPs to RCPs, and to further explore the possible applications of DHMA, the method was directly compared to a conventional turn-off approach. Increasing the amplification time from 10 to 60 min only doubles the number of MNPs per RCP. Therefore, a long amplification time is not necessarily required for improving the limit of detection in the assay. The turn-on approach based on DHMA provides critical information for improving the assay performance and understanding the underlying microscopic binding process.

## 2. Results

### 2.1. Effect of Amplification Time

To investigate the binding kinetics, four different rolling circle amplification times of 10, 20, 40 and 60 min and two RCP concentrations of 1.13 and 11.3 pM labelled with 250 μg/mL (250 pM equivalent) magnetic markers for analysis were used. The MNP markers are incubated with RCPs for 20 min at 55 °C and hybridization buffer is used for diluting the samples. The negative control (NC) sample consists of hybridization buffer and is mixed with the same amount of MNPs as in the test samples. It therefore contains no RCPs. The samples are then analyzed using both the turn-off and turn-on strategies.

#### 2.1.1. Turn-Off Analysis

**Figure 1:**
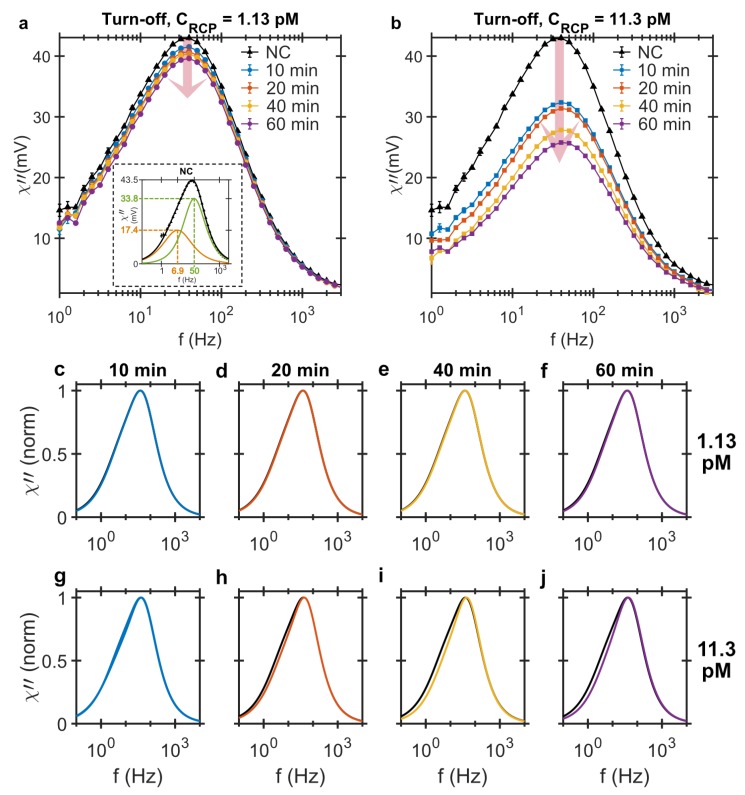
Imaginary component of ac magnetic susceptibility measured using the turn-off detection strategy versus the excitation frequency from MNP-labeled RCPs of (**a**) 1.13 pM and (**b**) 11.3 pM in concentration amplified for 10, 20, 40 and 60 min. The samples are diluted in hybridization buffer. The signals’ peak amplitudes decrease with increasing the RCP concentration and amplification time. The inset in (**a**) shows the bi-modal Cole-Cole model fitted to the imaginary component of the NC sample and shows that the MNP system has an intrinsic bi-modal size distribution with corresponding Brownian relaxation frequencies of 7 and 50 Hz; (**c**–**j**) Normalized bi-modal Cole-Cole model fitted to the imaginary component of ac susceptibility in the turn-off detection method for the magnetically labeled RCPs of (**c**–**f**) 1.13 pM and (**g**–**j**) 11.3 pM rolled for 10, 20, 40 and 60 min. The black curve that is present in all the panels corresponds to the bi-modal model fitted to the NC sample.

In the turn-off approach, the ac susceptibility of each sample is measured and compared with the NC sample. The drop in the peak imaginary ac susceptibility due to the presence of RCPs is the detection signal and is a function of the RCPs’ concentration. Figure 1a,b shows the imaginary part of the ac susceptibility as a function of the magnetic excitation field frequency for the different amplification times and RCP concentrations. The results show that increasing the amplification time results in a larger drop in the peak imaginary ac susceptibility signal indicating that there are more binding sites available for the MNPs.

Since the concentrations of RCPs (1.13 and 11.3 pM) and MNPs (250 pM) are known, the number of MNP-RCP complexes can be evaluated. The number of MNPs per RCP, *g*, is given by dividing the total number of RCPs, $C_{RCP}$, by the bound MNP markers, $C_{MNP}^{bnd}$. Assuming the magnetic moments of the MNPs do not change, the imaginary ac susceptibility signal is a measure of the MNP concentration and the amount of bound MNPs is a linear function of the decrease in the peak amplitude of the imaginary component [17]. Therefore, the number of MNPs per RCP is given by:

(1) $$g=\frac{C_{MNP}^{bnd}}{C_{RCP}}=\left(\frac{\chi {\prime \prime }_{NC}-\chi {\prime \prime }_{RCP}^{t}}{\chi {\prime \prime }_{NC}}\right)\times \frac{C_{MNP}}{C_{RCP}}$$

where $\chi {\prime \prime }_{NC}$ is the peak amplitude of the imaginary ac susceptibility from the NC sample, $C_{MNP}$ is the concentration of the MNPs in the sample, and $\chi {\prime \prime }_{RCP}^{t}$ is the peak amplitude of the imaginary ac susceptibility from the sample with concentration of $C_{RCP}$ and the amplification time *t*. Table 1 summarizes the estimated number of MNPs per RCP for each concentration of RCP and amplification time. As expected, the number increases with increasing amplification time; however, there are twice as many MNPs per RCP in the 1.13 pM sample compared to the 11.3 pM sample. This could be due to the higher number of available MNPs per RCP during the hybridization in the 1.13 pM sample compared to the 11.3 pM sample, $\frac{C_{MNP}^{NC}}{C_{RCP}}\approx$ 221 and 22.1 MNPs per RCP, respectively.

A bi-modal relaxation model based on a superposition of two Cole-Cole models [19] is used to fit the data. By normalizing the fitted bi-modal Cole-Cole model to the peak amplitude, we can visually compare the response curves of the turn-off detection for any change in the size distributions in the samples. We should emphasize that the MNP system also has an intrinsic asymmetric size distribution. The inset of Figure 1a shows the fitted bi-modal Cole-Cole model to the NC sample in hybridization buffer with the two distinct Brownian relaxation frequencies of 7 and 51 Hz. The asymmetrical size distribution of this particular particle system has also been previously observed elsewhere [17,19]. The intrinsic LRF of the NC should not be confused with the LRF from the MNP-RCP complexes. As we shall see later, the DHMA allows distinction of the LRFs from the NC and the MNP-RCP complexes.

Figure 1c–j shows the normalized Cole-Cole plots for all samples measured with the turn-off strategy. The black curve, which is present in all the plots, is the bi-modal Cole-Cole model fitted to the NC sample, also normalized to its peak amplitude. For the 1.13 pM samples, c.f. Figure 1c–f, the response signals are nearly identical to the NC sample irrespective of the amplification time. However, for the 11.3 pM samples, Figure 1g–j, the contribution from the LRF becomes slightly more visible with increasing amplification time. Also, at such high concentrations of RCP, MNPs bind to several RCPs at the same time, creating large agglomerates that sediment and completely disappear from the measurement frequency window. The sedimentation of the agglomerates are visible to the bare eye after hybridization and is also reported elsewhere [28,32].

#### 2.1.2. DHMA, Turn-On Analysis

**Figure 2:**
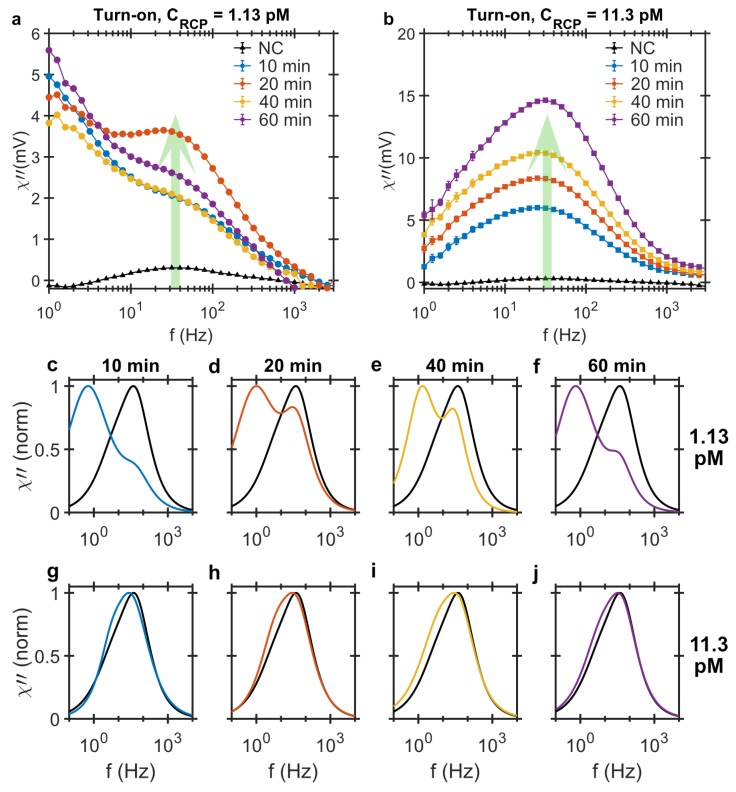
Imaginary component of ac magnetic susceptibility measured using the turn-on detection strategy versus the excitation frequency from MNP-labeled RCPs of (**a**) 1.13 pM and (**b**) 11.3 pM in concentration amplified for 10, 20, 40 and 60 min. The dilution samples are prepared using hybridization buffer. All the samples are measured using the NC as the control. The detection signal in this case increases with increasing RCP concentration and amplification time. The LRF (<15 Hz) distribution at low concentration is distinctly present in the turn-on measurement strategy; (**c**–**j**) Normalized bi-modal Cole-Cole model fitted to the imaginary component of turn-on strategy for the MNP-labeled RCPs of (**c**–**f**) 1.13 pM and (**g**–**j**) 11.3 pM rolled for 10, 20, 40 and 60 min. The black curve that is present in all the panels corresponds to the bi-modal model fitted to the NC sample.

For the turn-on analysis, we use the DHMA method [31] which measures a test sample and a reference sample simultaneously. The reference sample in this case is the NC and it fills one of the two available microfluidic channels. The second channel is filled with a test sample containing RCPs which are amplified for 10, 20, 40 and 60 min and have different concentrations of 1.13 pM and 11.3 pM. The magnetic signals from the samples resting in the two microfluidic channels are measured with a gradiometer sensor where the they are subtracted and only the difference in two signals is read out, Figure 2a,b. For 1.13 pM samples, the LRF contribution, which is the result of MNP-RCP complex formation, is rather large, resulting in a distinct bi-modal response signal. For the RCP concentration of 11.3 pM, the populations of MNP-RCP complexes in the test sample and the corresponding unbound MNPs in the control sample are larger which results in a broad detection signal with the peak position shifted to lower frequency. The larger size of the MNP-RCP complexes compared to unbound MNPs in the ensemble shifts the peak position to lower frequencies. Increasing the amplification time results in larger RCPs which at such high RCP concentration form agglomerates when hybridizing with MNPs. The large agglomerates have their relaxation frequency out of the measurement frequency window, thereby bringing the frequencies of the two peak position back to the NC sample value again.

To visualize the bi-modal response signals and frequency shifts, the data is fitted to the bi-modal Cole-Cole model. The model provides a better picture of the underlying dynamic process. Bi-modal Cole-Cole distributions fitted to the differential signals and normalized to the peak amplitudes are plotted in Figure 2c–j and the fitting parameters are listed in Table 1. One important fitting parameter is $\frac{\chi_{01}}{\chi_{02}}$, c.f. Equation (4), which gives the ratio of LRF and HRF contributions in the total bi-modal distribution of the response signal. The results show that for the samples with 1.13 pM concentration, the LRF position is below 1.5 Hz and the contribution of the LRF is larger than the HRF, meaning $\frac{\chi_{01}}{\chi_{02}}$ is larger than one. The HRF moves to lower frequencies with increasing the amplification time which indicates that the MNP-RCP complexes are larger. The shift to lower frequencies is also visible for samples of 11.3 pM RCP concentrations; however, it is more pronounced at shorter amplification times where the contribution of the two distributions are very close, thereby shifting the peak frequency position below that of the NC sample. The shape and position of the peaks approach those of the NC sample with increasing amplification time, c.f. Figure 2g–j. The loss of LRF distribution here is also due to the agglomeration of the MNP-RCP complexes. These agglomerates sediment in the sample vials and are visible to the bare eye and therefore their relaxation frequency is out of the measurement frequency window.

### 2.2. Effect of RCP Concentration in Turn-On Analysis

**Figure 3:**
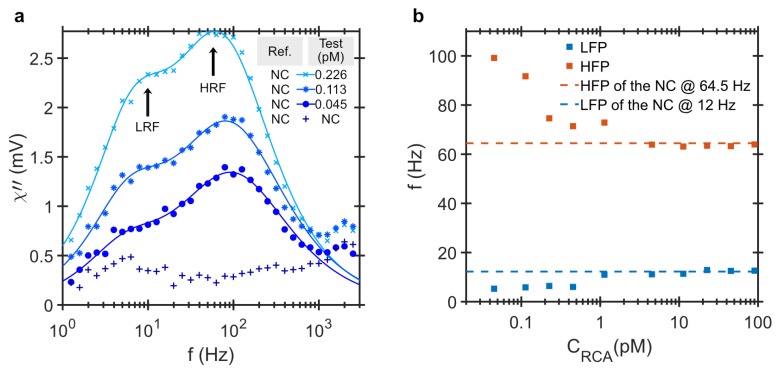
The binding process between MNPs and RCPs rolled for 20 min at low RCP concentrations. (**a**) Imaginary component of differential ac susceptibility versus the frequency for three RCP concentrations, 45 fM, 113 fM and 226 fM diluted in PBS buffer which results in slightly higher values for LRF and HRF compared to samples presented in Figure 1 and Figure 2 due to lower viscosity of the buffer. The lines are the result of fitting to a bi-modal relaxation model. There are two frequency components visible in the imaginary part of the differential ac susceptibility and they are defined as the low relaxation frequency peak (LRF) and the high relaxation frequency peak (HRF); (**b**) The LRF and HRF are extracted from fitting the bi-modal relaxation model to the imaginary component of the differential ac susceptibility versus the RCP concentration. For high RCP concentration, the values of both the LRF and HRF are shifted to 12 and 64.5 Hz, respectively. These two frequencies corresponds to the LRF and HRF of the intrinsic bi-modal distribution of the NC sample in PBS buffer.

To study the effect of the RCP concentration, the DHMA signal is measured for RCPs amplified for 20 min and concentration ranging from 45 fM to 90 pM. Figure 3a shows the imaginary part of the differential ac susceptibility as a function of frequency for the NC sample and three positive test samples with the low RCP concentrations of 45 fM, 113 fM and 226 fM. Two peaks become prominent with increasing RCP concentration. The LRF peak corresponds to the Brownian relaxation of the MNP-RCP complex in the positive test sample and the HRF peak is due to unbound MNPs and its magnitude corresponds to the relative difference between unbound MNPs in the test and the NC samples. Figure 3b shows the frequencies of the LRF and HRF as a function of RCP concentration extracted by fitting the imaginary part of the differential ac susceptibility using a bi-modal Brownian relaxation model. At high RCP concentrations, the LRF and the HRF saturate at 12 and 64.5 Hz, respectively. These frequencies correspond to a characteristic bi-modal distribution of the functionalized MNPs system in PBS buffer. The ionic strength of the buffer affects the electrostatic interaction in the solution and changes its viscosity [33] and may even affect the size distribution of the MNP system [34]. The PBS buffer, in this case, has a lower viscosity compared to the hybridization buffer due to less salt concentration. Therefore, the values of the LRF and HRF ’of the NC are higher than the ones in Figure 1 and Figure 2 which are listed in Table 1. At low RCP concentrations, the HRFs are at higher frequencies than that of the NC. This implies that the smaller particles in the MNP size distribution hybridizes to the RCPs first. We attribute this to the larger diffusion coefficient of the smaller particles in the MNP distribution. The diffusion coefficient of a particle in the solution is inversely related to its size and is given by Equation (2). Considering the large intermolecular distances at these low concentrations, it is thus more likely for smaller MNPs to meet the RCPs and thus take precedence in binding during the hybridization process. The MNP system has a log-normal size distribution with a median hydrodynamic size of 100 nm. Those MNPs with hydrodynamic sizes smaller than the median 100 nm thus comprise only a small part of the entire MNP population.

**Figure 4:**
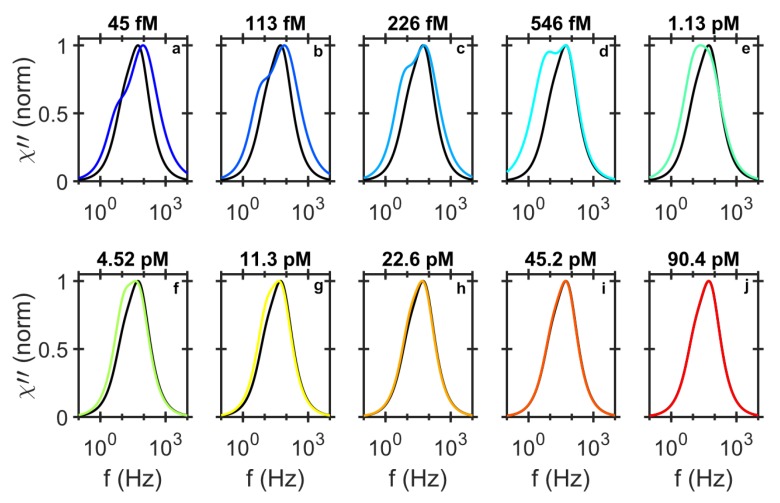
Normalized imaginary component of the differential ac susceptibility. To compare the shape of the imaginary component of the differential ac susceptibility from different RCP concentrations diluted in PBS (**a**–**j**), we normalized the fitted bi-modal model to the imaginary responses and plotted them individually with the normalized imaginary response from the NC sample (black line). The shapes of the response curves, especially at low concentration of the RCPs are very different form the NC sample. For higher concentration of RCPs, the low frequency contribution decreases and falls outside of the measurement frequency window and the shape of the response signal becomes similar to the NC sample.

As the RCP concentration increases and the small MNPs in the size distribution are all bound, the larger MNPs start to hybridize to the RCPs. This results in a shift in the HRF from 100 Hz to 64.5 Hz. At low concentration of RCPs, the LRF peak position appears at approximately 5 Hz, which corresponds to the average size of the MNP-RCP complexes, ∼500 nm. With increasing RCP concentration, the LRF amplitude increases, as more MNP-RCP complex form. The LRF finally slowly shifts to 12 Hz, which is the LRF of the MNPs. This behaviour is illustrated in Figure 4 where the shape of the amplitude normalized distribution for different RCP concentrations are compared. We have previously shown that for this assay the equilibrium dissociation constant is $K_{D}=16.5$ pM [31]. The equilibrium dissociation constant is a measure of tendency for the MNP-RCP complex to dissociate into separate MNP and RCP entities. Also, when the concentration of the RCPs is equal to $K_{D}$, half of the MNP markers are consumed. When the concentration of the RCPs is close to or larger than $K_{D}$, it is more likely for a single MNP to conjugate to two or more RCPs and thus form an agglomerate. The agglomerates can become very large and sedimented, which can be even visible to the bare eye. The Brownian relaxation frequency of agglomerates falls outside of the measurement frequency window which shifts the LRF to that of the NC, i.e., 12 Hz. When all MNPs in a positive test sample are effectively immobilized, the shape of the differential readout signal becomes similar to the one from the NC sample, see Figure 4g–j.

### 2.3. Nanoparticle Tracking Analysis

**Figure 5:**
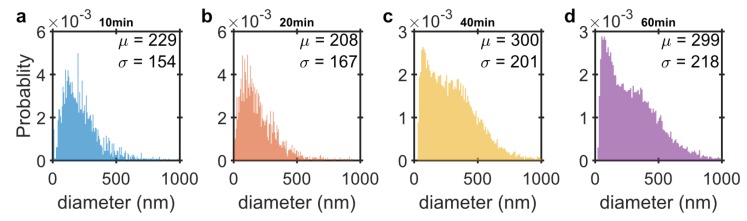
NTA number-weighted probability size distribution of the (**a**) 10; (**b**) 20; (**c**) 40 and (**d**) 60 min rolled RCPs labelled with fluorescent marker. The mean hydrodynamic diameter, μ, and standard deviation, σ, are obtained from fitting a normal distribution to the data.

To obtain the size distribution of the RCPs for each amplification time, NTA is employed. The RCPs in this case are only tagged with fluorescent markers to obtain a more precise size distribution. Unlike the MNPs that have multiple oligonucleotides covering their surface and may hybridize to more than one RCP, the fluorescent tagged oligonucleotides can only bind to one RCP and therefore, they do not induce any agglomeration. Figure 5 shows the size distribution for RCPs with 10, 20, 40 and 60 min amplification times obtained from NTA measurements. The mean and standard deviation is determined from fitting a normal distribution to the measurements. The mean hydrodynamic diameter of the RCPs increases only slightly with increasing amplification time. The RCPs with a longer amplification time, however, have a greater dispersion in their hydrodynamic size distribution compared to shorter amplification times. The RCPs are diluted in the hybridization buffer for NTA measurements. The high electrolyte concentration in the buffer makes the RCPs which are DNA coils more compact and interwound by overcoming the electrostatic repulsion from the negatively charged backbone of the DNA [35]. The high salt concentration in the buffer make the RCPs to fluoresce as a single point object which is necessary for tracking the RCPs in the NTA measurements.

## 3. Discussion

Figure 1c–f compares the Cole-Cole fits to the data from turn-off strategy for test samples of 1.13 pM RCP concentration and different amplification times and it reveals no visible change in the MNP size distributions. The fit to the test samples entirely overlaps the NC sample. This is also true even when increasing the RCP concentrations to 11.3 pM, c.f. Figure 1g–j. The LRF and HRF values extracted for both 1.13 and 11.3 pM RCP concentrations and all four amplification times are the same as NC sample, i.e., 7 and 51 Hz. The $\frac{\chi_{01}}{\chi_{02}}$ parameter given in Table 1, which gives the contribution of the LRF in the total bi-modal distribution, is almost constant and only changes around 20–30% for the longest amplification time and 11.3 pM concentration of RCPs. Since the variations in fitting parameters for different NC sample are also around 15%, these changes can be considered insignificant. The values of the LRF and HRF extracted from all the test samples are also the same as the ones for the NC sample and therefore, provides no further information about the binding characteristics. The main issue with this approach is the magnetic background from the unbound MNP markers in the solution.

In the turn-on analysis using the DHMA, however, both the NC sample and the test sample containing the RCPs are measured simultaneously and their corresponding ac susceptibility signals subtracted at the sensor. The difference in the particle size distribution of the two samples is measured and thus, the magnetic background from the excess unbound MNPs in the test sample is eliminated by the NC sample. The DHMA thus measures the magnetic ac susceptibility from the combination of: (I) the MNP-RCP complexes in the test sample and (II) the excess MNPs in the NC sample which is a result of MNPs hybridized to RCPs in the test sample forming the MNP-RCP complexes. The outcome of this analysis is an assay that is sensitive to the low concentrations of the RCPs and the signature of the MNP-RCP complex is directly measured in this readout approach. Normalized Cole-Cole fits to the 1.13 pM RCP concentration, c.f. Figure 2c–f, show a double peak distribution and a clear deviation from the NC response. The LRF peak is related to the MNP-RCP complexes and the HRF to the excess markers in the NC sample. As the $\frac{\chi_{01}}{\chi_{02}}$ parameter is larger than 1, the LRF contribution has a larger contribution in the bi-modal distribution.

Furthermore, the LRF and HRF peak positions in the DHMA signal depend on the RCP concentration, c.f. Figure 4, therefore, the whole frequency window especially at low frequencies should be used as a detection signal. There are two distinct processes that influence the LRF and HRF peak positions. First, the binding of the MNPs with RCPs and the formation of individual MNP-RCP complexes. This gives rise to the LRF peak which appears at low frequencies due to larger hydrodynamic size of the complex compared to the free MNPs. The LRF peak amplitude gradually increases with increasing the RCP concentration. At 1.13 pM, the LRF peak amplitude is larger than the HRF peak amplitude. This is the point where the $\frac{\chi_{01}}{\chi_{02}}$ ratio from the bi-modal Cole-Cole analysis reaches a maximum value of ≈1.43. Increasing the concentration of the RCP further, cross-linking between the individual MNP-RCP complexes starts. Since these cross-linked complexes are very large (they sediment in the sample solutions and are visible by bare eye), the $\frac{\chi_{01}}{\chi_{02}}$ ratio decreases for increasing the RCP concentration, and the LRF and HRF saturate to those of the NC at the RCP concentration of 90.4 pM. This implies that the LRF contribution diminishes with increasing RCP concentration due to the formation of the agglomerates (cross-linked MNP-RCP complex) which have Brownian relaxation frequencies outside our measurement window.

The estimated number of MNPs per RCP, *g*, contains an important information that helps optimize the magnetic assay. Unlike the similar RCA assays, which are based on fluorescent markers, increasing the amplification time does not drastically increase the number of magnetic markers per RCP. This can be explained by the fact that the size distribution of the RCPs does not change very much with amplification time. This is supported by the NTA measurements that reveal rather broad size distributions for all amplification times. It also shows that the mean hydrodynamic size increases only slightly with the amplification time. This is also in agreement with the observation that the LRF does not change with the amplification time, See Figure 2a. The broad size distribution of the RCPs have also been observed by opening and stretching the RCPs both on a glass and by confinement of DNA in nanochannels [36].

Comparing the *g* parameter for the 1.13 pM and 11.3 pM RCP concentrations also indicates that a higher ratio of MNP per RCP in solution helps increase the number of bound MNPs per RCP. This means that the assay performs best when the number of markers is in excess of the RCPs. An apparent way to ensure that, is to use a higher concentration of MNP markers in the assay. That is, however, unfavourable for the turn-off detection approach as it becomes challenging to resolve the small detection signal in a presence of a large background. This large background is nevertheless eliminated in the differential analysis. The advantage of increasing the markers concentration is faster binding kinetics, especially at low concentration of RCPs.

The turn-on strategy using the DHMA can clearly distinguish the LRF and HRF peaks corresponding to the MNP-RCP complexes and the unbound MNPs even at very low concentrations of the RCPs. This is a crucial piece of information which is not available in turn-off detection approaches. Furthermore, we observe a preferential binding of the smaller MNPs to RCPs at low concentrations of RCPs and also formation of agglomerates due cross-linking of individual MNP-RCP complexes at high RCP concentrations. Increasing the amplification time does not significantly increase the number of MNPs bound to each individual RCP which is important for optimization of the magnetic assay and increasing its sensitivity to target analyte. This has also been confirmed by the NTA measurements. The zero starting signal in the DHMA makes the quantification of MNP-labeled RCPs easier and provides us with a powerful technique for dynamically characterizing the binding interactions between MNPs and biomolecules in fluid volumes. The LRF peaks can be monitored over time during the hybridization process to provide time dependent analysis. By incorporating a heater in the PDMS chip and sweeping the temperature through the melting point of the MNP-RCP binding, the binding/releasing of the MNPs to/from RCPs can be observed over time. The performance of the biosensor can also be improved by fully implementing the nucleic acid assay on a lab-on-a-chip.

## 4. Materials And Methods

### 4.1. Rolling Circle Amplification

Vibrio cholerae synthesized target DNA (Biomers, Ulm, Germany) is hybridized to the padlock probe and ligated forming a circle DNA. The padlock probe is a linear molecule with target complementary ends. To perform this, a ligation mixture of 5 μL of 10 ×ϕ29 buffer, 2.5μL ATP (20 mM), 1μL padlock probe (1μM), 3μL target DNA (1μM), 1μL T4 ligase (1 U/μL) and 37.5μL Milli-Q water is prepared and incubated at 37 °C for 15 min. The circular padlock probes are then amplified for different times using the RCA method to form RCPs of different sizes. The RCA mixture consists of 25μL of the ligation mix prepared earlier, 6μL of 10 ×ϕ29 buffer, 4μL dNTP (2.5 mM), 6μL BSA (2μg/μL), 0.4μL of ϕ29 polymerase (10 U/μL), and 18.6μL of Milli-Q water. Four RCA mixtures are prepared and incubated at 37 °C for 10, 20, 40 and 60 min followed by 5 min of enzyme inactivation at 65 °C. To obtain a solution containing 5 nM RCPs (based on the initial padlock probe concentration), 40μL of hybridization buffer containing 0.1 M EDTA, 0.1 M Tris-HCl (pH 8), 0.005% Tween 20, and 2.5 M NaCl is added to the mixtures. Considering the 22.6% success rate of the assay [37], the effective RCP concentration of the mixtures are 1.13 nM instead of 5 nM. By further diluting the original RCP mixture with hybridization buffer, we prepare different concentrations of RCPs for the experiments. Table 2 lists the sequences for the target, padlock probe and the detection oligonucleotide.

### 4.2. Labelling The Rcps

In this study, we labeled the RCPs with fluorescent marker of atto-488 or magnetic markers consisting of magnetic nanoparticle. The atto-488 are attached to the 3’ side of the detection oligonucleotide and have their excitation wavelength in the range of the laser wavelength used in our NTA setup. The MNP system used, according to its datasheet, is a suspended iron oxide-based streptavidin coated multi-core particles with median diameter of 100 nm for the core (micromod Partikeltechnologie GmbH, Rostock, Germany). The detection oligonucleotides also have a biotinylated side which can bind to the streptavidin coating of the MNPs and functionalizing them for specific binding to the RCPs. To do so, the MNPs (10 mg/mL) are washed twice with 1 × Wtw buffer (10 mM Tris-HCl, 5 mM EDTA, 0.1% Tween 20, 0.1 M NaCl) using a permanent magnet and thereafter re-suspended in the 1 × Wtw buffer and incubated with 10μM biotin-conjugated oligonucleotides for 30 min at room temperature. After incubation, the MNPs are washed again twice with the 1 × Wtw buffer and suspended in phosphate-buffered saline (PBS) in its original volume. To conjugate the fluorescent or the magnetic markers to the RCPs, either of the two markers and the RCPs are mixed and the solution incubated for 20 min at 55 °C. Different concentrations are prepared by diluting the mixture in PBS or hybridization buffer.

### 4.3. Nanoparticles Tracking Analysis

Nanoparticle tracking analysis (NTA) is a technique that uses either the light scattering or fluorescence signal to track Brownian motion from which it can determine the size distribution of samples in liquid suspension. A laser beam of specific wavelength is passed into the sample chamber containing the nanoparticle suspension with reduced profile [38]. The particles in the path of the beam then scatter or absorb the light which is easily visible using a low magnification microscope objective. A CMOS camera records the particles movement in the fields of view. A software then identifies each particle and tracks its movements under Brownian motion on a frame by frame basis and calculates the average distance moved by the particle. The spherical equivalent of the particle diameter, *d*, is given by the Stokes-Einstein equation

(2) D=kT/3πηd

where *D* is the particle diffusion coefficient, *k* is the Boltzman’s constant and *T* is the temperature. The NTA measurements were performed with a NanoSight LM20 (Nanosight, Amesbury, UK) which uses a 488-nm laser. The samples were injected in the sample chamber using a sterile syringe and a syringe pump. All measurements are performed at room temperature and samples were measured three times for 180 s with manual shutter and gain adjustment.

### 4.4. Magnetic Ac Susceptibility Analysis

For the studied 100 nm magnetic particle system, the Brownian relaxation time is shorter than Néel relaxation and therefore, the effective relaxation process associated with this particle system is dominated by the Brownian rotational motion. The Brownian relaxation time, $\tau_{B}$, and frequency, $f_{B}$, of the MNP ensemble is given by

(3) $$\tau_{B}=1/\left(2\pi f_{B}\right)=3\eta V_{B}/kT$$

where η is the viscosity of the solvent, *k* is the Boltzman’s constant, *T* is the temperature and $V_{B}$ is the hydrodynamic volume of the particle. The MNPs are functionalized with fluorescent marked biotinylated oligonucleotide strands for hybridization with the RCPs.

The dynamic magnetic properties of the suspended MNP ensemble are detected using magnetic ac susceptibility. An ac magnetic field in the range of 1–3000 Hz is applied to the sample and both the in-phase and out-of-phase components of the magnetic response of the solution is measured using first order planar high transition superconductor gradiometer loop coupled to a high-$T_{c}$ superconducting quantum interference device (SQUID). The in-phase and out-of-phase response of the sample correspond to the real and imaginary components of the complex susceptibility. The actual complex susceptibility is a calibration factor that converts the voltage output of the sensor to the sample magnetic moment. Therefore, the susceptibility signals are expressed in voltage.

Here we use two strategies for detection of target molecules using the ac susceptibility analysis, turn-off and turn-on (or differential) strategies. In the turn-off detection, a NC samples is measured, and the peak amplitude of imaginary component is taken as a reference point. Upon binding of the MNPs to the target molecules, the magnitude of the peak amplitude of the imaginary component of the ac susceptibility decreases which is taken as the detection signal [9,17,27]. The differential ac susceptibility takes advantage of the symmetry in the gradiometer sensor to measure the relative difference in the ac susceptibility responses of two suspended magnetic samples. The magnetic flux from each sample is coupled to an individual loop of the gradiometer using two identical microfluidic channels that are aligned parallel to the baseline of the gradiometer. The screening current in each loop is then subtracted in the middle line of the loop measured by the SQUID providing the relative difference in the ac susceptibility of the two samples [31]. A bi-modal relaxation model based on a superposition of two Cole-Cole models [19]:

(4) $$\chi \left(\omega \right)=\frac{\chi_{01}}{1+{\left(i\omega \tau_{1}\right)}^{\alpha_{1}}}+\frac{\chi_{02}}{1+{\left(i\omega \tau_{2}\right)}^{\alpha_{2}}}+\chi_{high}$$

is used to fit to the data presented here. $\chi_{01}$ and $\chi_{02}$ are the DC susceptibilities, $\chi_{high}$ is the high frequency contribution to the real part of the susceptibility, $\tau_{1}$ and $\tau_{2}$ are the relaxation time and $\alpha_{1}$ and $\alpha_{2}$ are the phenomenological relaxation distribution parameters for the 1st and 2nd relaxation modes, respectively. The resulting frequencies are extracted from $\tau_{1}$ and $\tau_{2}$ and are related to the HRF and LRF from the unbound MNPs and the MNP-RCP complexes, respectively.

## 5. Patents

S.S., A.K., and D.W. are co-inventors of a patent filed at the Swedish patent and registration office, patent pending 1950159-2(2019), on the DHMA.

## Figures and Tables

**Table 1:** Estimated number of MNP markers per RCPs, *g*, for each amplification time and RCP concentration calculated using Equation (1) and the bi-modal Cole-Cole fitting parameters for the turn-off and turn-on (DHMA) analysis presented in Figure 1 and Figure 2.

Measurement Method	Concentration	Parameters	Amplification Times				
			NC	10 min	20 min	40 min	60 min
Turn-off Analysis	1.13 pM	***g***	–	8	12	14	18
		$\frac{\chi_{01}}{\chi_{02}}$	0.65	0.61	0.60	0.61	0.55
		LRF (Hz)	6.9	6.9	6.9	6.9	6.9
		HRF (Hz)	50.0	50.3	50.2	50.2	50.1
	11.3 pM	***g***	–	5	6	8	9
		$\frac{\chi_{01}}{\chi_{02}}$	0.73	0.64	0.56	0.47	0.52
		LRF (Hz)	6.9	6.0	6.9	6.9	6.7
		HRF (Hz)	52.4	51.8	52.9	52.6	51.3
Turn-on Analysis	1.13 pM	***g***	–	11	14	14	20
		$\frac{\chi_{01}}{\chi_{02}}$	0.65	5.94	2.09	1.74	4.87
		LRF (Hz)	7.0	0.57	0.85	1.33	0.63
		HRF (Hz)	51.0	51.66	41.92	30.61	40.78
	11.3 pM	***g***	–	3	5	6	8
		$\frac{\chi_{01}}{\chi_{02}}$	0.73	0.25	0.73	1.48	1.04
		LRF (Hz)	7.0	4.91	5.82	6.87	6.88
		HRF (Hz)	52.0	34.05	45.82	52.10	52.40

**Table 2:** DNA sequences of target, padlock probe and detection oligonucleotide for rolling circle amplification of Vibrio Cholerae. The padlock probe has motifs that matches the target Cholera sequence (green and blue) and forms a circle by hybridizing to it. The detection oligonucleotide is part of the padlock probe (red) and therefore, hybridizes with the RCP of the circularized padlock probe.

Oligonucleotides	Sequences from 5’ to 3’
Target	CCCTGGGCTCAACCTA GGAATCGCATTTG
Padlock probe	TAGGTTGAGCCCAGGG ACTTCTAGAGTGTACCGACCTCAGTAGCCGTGACTATCGACTT GTTGATGTCATGTGTCGCAC CAAATGCGATTCC
Detection oligonucleotide	biotin-TTTTTTTTTTTTTTTTTTTT GTTGATGTCATGTGTCGCAC- atto488

## Supplementary Materials

The following are available online at https://www.mdpi.com/2079-6374/9/3/109/s1, Figure S1: (a) A schematic diagram of the DHMA, (b) turn-off detection approach (c) turn-on detection approach; Figure S2: Turn-on detection signal versus the RCP concentration.

## Abbreviations

The following abbreviations are used in this manuscript:

MNP	magnetic nanoparticle
RCA	rolling circle amplification
RCP	rolling circle product
DHMA	differential homogenous magnetic assay
SQUID	superconducting quantum interference device
NTA	nanoparticle tracking analysis
HRF	high relaxation frequency
LRF	low relaxation frequency
