# Supplementary material for: Characterization of Binding of Magnetic Nanoparticles to Rolling Circle Amplification Products by Turn-On Magnetic Assay
Source: Biosensors (Basel). 2019 Sep 17;9(3):109. doi: 10.3390/bios9030109 (PMC6784358; doi:10.3390/bios9030109)
Supplement: Supplementary file 1 [file biosensors-09-00109-s001.pdf]

Supplementary material

Characterization of binding of magnetic nanoparticles to rolling circle amplification products by turn-on magnetic assay

Sobhan Sepehri [1*], Björn Agnarsson [2], Teresa Zardán Gómez de la Torre [3], Justin F. Schneiderman [1,4], Jakob Blomgren [5], Aldo Jesorka [6], Christer Johansson [5], Mats Nilsson [7], Jan Albert [8,9], Maria Strømme [3], Dag Winkler [1], and Alexei Kalaboukhov [1]

[1] Department of Microtechnology and Nanoscience – MC2, Chalmers University of Technology, SE-412 96 Göteborg, Sweden;
[2] Department of Physics, Chalmers University of Technology, SE-412 96 Göteborg, Sweden;
[3] Department of Engineering Sciences, Uppsala University, The Ångström Laboratory, Box 534, SE-751 21 Uppsala, Sweden;
[4] MedTech West and the Institute of Neuroscience and Physiology, University of Gothenburg, SE-405 30 Göteborg, Sweden;
[5] RISE – Research Institutes of Sweden, SE-411 33 Göteborg, Sweden
[6] Department of Chemistry and Chemical Engineering, Chalmers University of Technology, SE-412 96 Göteborg, Sweden;
[7] Science for Life Laboratory, Department of Biochemistry and Biophysics, Stockholm University, Box 1031, SE-171 21 Solna, Sweden;
[8] Department of Clinical Microbiology, Karolinska University Hospital, SE-171 76 Stockholm, Sweden;
[9] Department of Microbiology, Tumor and Cell Biology, Karolinska Institute, SE-171 77 Stockholm, Sweden;
* Correspondence: sobhan.sepehri@chalmers.se

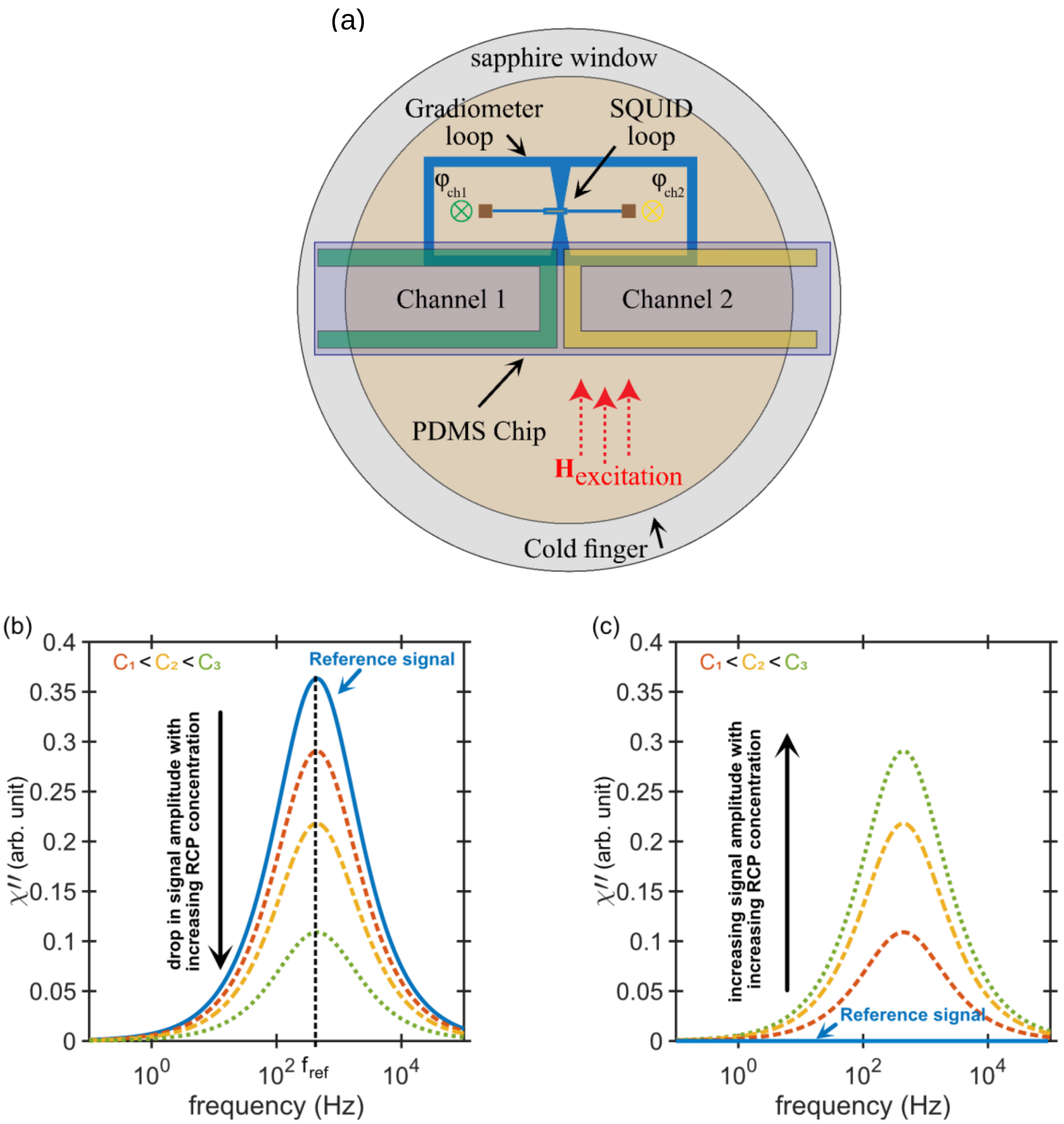

Fig. S1. (a) The schematic diagram of the differential ac susceptibility measurement setup using a high temperature superconducting SQUID gradiometer sensor and two microfluidic channels embedded in a single PDMS chip. Having the excitation magnetic field in plane and perpendicular to the baseline of the gradiometer, the magnetic samples in Cha. 1 and Cha. 2 of the chip are coupled to individual pickup loops of the gradiometer sensor. (b) Imaginary component of the ac susceptibility as a function of the external magnetic field frequency in a turn-off detection approach. This approach only uses one of the two available channels for the measurements. The channel is first filled with NC sample to measure the reference signal which maximizes at the Brownian relaxation frequency of the MNP labels, f_{ref}. Introducing the positive test sample containing the RCPs, the amplitude of the signal at f_{ref} decreases due to binding of the MNPs with RCPs. The extinction of the signal increases with increasing RCP concentration in the positive test samples. (c) In the turn-on detection approach using the DHMA, the two channels are filled. The test samples in this detection approach are always measured while having the NC sample in the other channel and therefore the difference in the ac susceptibility of the two samples is measured. Therefore, having the NC sample as the test sample results in a zero-response signal which is taken as a reference. The detection signal then builds up upon increasing the concentration of the RCPs in the positive test samples. This is due to hybridization of the MNP probes in the positive test sample by RCPs causing a difference in the MNP concentration and size distribution in the two samples.

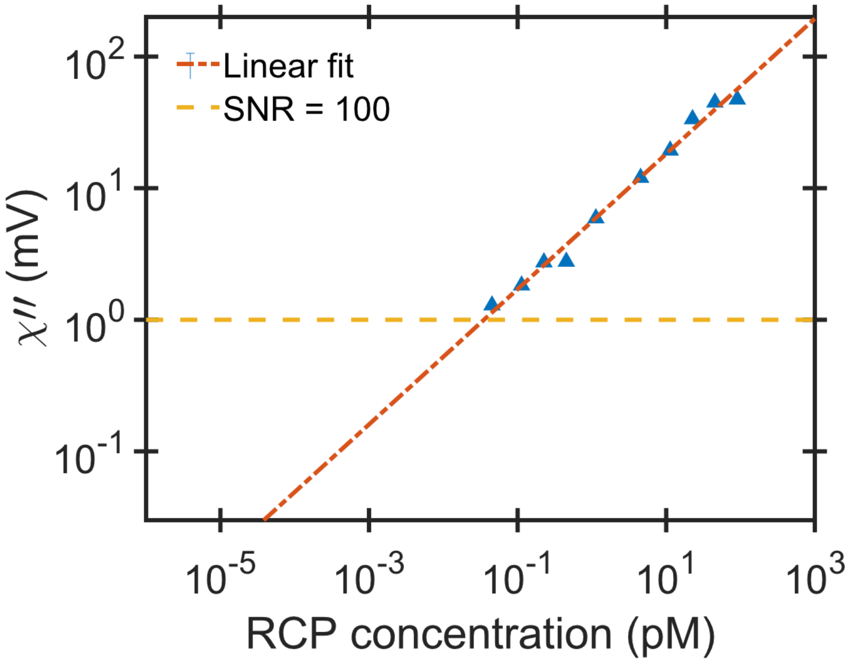

Fig. S2. Peak amplitude of the imaginary component of the ac susceptibility at 65 Hz in the turn-on detection approach versus the RCP concentration in the positive test sample amplified for 20 min[1].

[1] Sepehri, S.; Zardán Gómez de la Torre, T.; Schneiderman, J.; Blomgren, J.; Jesorka, A.; Johansson, C.; Nilsson, M.; Albert, J.; Strømme, M.; Winkler, D.; Kalaboukhov, A. Homogeneous differential magnetic assay. ACS Sensors 2019, p. acssensors.9b00969. doi:10.1021/acssensors.9b00969.
